# Image-guided robotic radiosurgery for the treatment of arteriovenous malformations

**DOI:** 10.1371/journal.pone.0266744

**Published:** 2022-09-22

**Authors:** Franziska Loebel, Antonio Pontoriero, Anne Kluge, Giuseppe Iatì, Gueliz Acker, Markus Kufeld, Alberto Cacciola, Stefano Pergolizzi, Sergio Vinci, Sara Lillo, Ran Xu, Carmen Stromberger, Volker Budach, Peter Vajkoczy, Carolin Senger, Alfredo Conti

**Affiliations:** 1 Department of Neurosurgery, Charité Universitaetsmedizin Berlin, Berlin, Germany; 2 Department of Radiation Oncology, Charité Universitaetsmedizin Berlin, Berlin, Germany; 3 Department of Radiation Oncology, University of Messina, Messina, Italy; 4 Department of Neuroradiology, University of Messina, Messina, Italy; 5 Department of Neurosurgery, Alma Mater Studiorum University of Bologna, Bologna, Italy; 6 IRCCS ISNB-Istituto delle Scienze Neurologiche Bologna, Bologna, Italy; Stanford University School of Medicine, UNITED STATES

## Abstract

**Background:**

Cerebral arteriovenous malformations (AVMs) are challenging lesions, often requiring multimodal interventions; however, data on the efficacy of stereotactic radiosurgery for cerebral AVMs are limited. This study aimed to evaluate the clinical and radiographic results following robotic radiosurgery, alone or in combination with endovascular treatment, and to investigate factors associated with obliteration and complications in patients with AVM.

**Methods:**

We retrospectively analyzed the clinical and imaging characteristics of 123 patients with AVMs of all Spetzler-Martin grades treated at two institutions by robotic radiosurgery in single-fraction doses (CyberKnife). Embolization was performed before radiosurgery in a subset of patients to attempt to downgrade the lesions. Factors associated with AVM obliteration and complications (toxicity) were identified via univariate and multivariate analyses.

**Results:**

The median follow-up time was 48.1 months (range, 3.6–123 months). Five patients were lost to follow-up. The obliteration rate in the 59 patients with a follow-up period exceeding four years was 72.8%. Complete obliteration and partial remission were achieved in 67 (56.8%) and 31 (26.3%) cases, respectively, whereas no change was observed in 20 cases (17.8%). Embolization was performed in 54/123 cases (43.9%). Complete and partial obliteration were achieved in 29 (55.7%) and 14 (26.9%) embolized patients, respectively. In the multivariate analysis, the factors associated with obliteration were age (p = .018) and the Spetzler-Martin grade (p = .041). Treatment-induced toxicity (radiation necrosis and/or edema) was observed in 15 cases (12.7%), rebleeding occurred in three cases (2.5%), and the rate of mortality associated with rebleeding was 1.7%.

**Conclusions:**

CyberKnife radiosurgery is a valid approach for treating AVMs of all Spetzler-Martin-grades, with satisfactory obliteration rates, low toxicity, and a relatively rare incidence of rebleeding.

## Introduction

Cerebral arteriovenous malformations (AVMs) are complex neurovascular lesions exhibiting an abnormal communication between arterial feeders and draining veins. Due to abnormal vascular structure and altered blood flow dynamics, there is an increased risk of intracranial hemorrhage, potentially leading to substantial neurological morbidity and mortality at rates of 2–4% per year [1–3]. AVM treatment remains controversial, and individualized treatment strategies comprise microsurgical resection, endovascular treatment, and/or stereotactic radiosurgery (SRS), in addition to watchful waiting [4]. Complete obliteration is the only strategy to abrogate the risk of AVM bleeding. While small AVMs in non-eloquent brain areas are best managed with complete microsurgical resection, more complex malformations (those with a grade of IV–V according to the Spetzler-Martin (SM) classification [5] are usually considered inoperable due to their eloquent localization or deep venous drainage. SRS is an established alternative strategy for treating such lesions [6–8]. By inducing progressive vascular injury, inflammation, and thrombosis, high-dose SRS of the nidus eventually leads to endoluminal obliteration following a latency period of several months to years, during which patients remain at risk for AVM hemorrhage.

**Fig 1:**
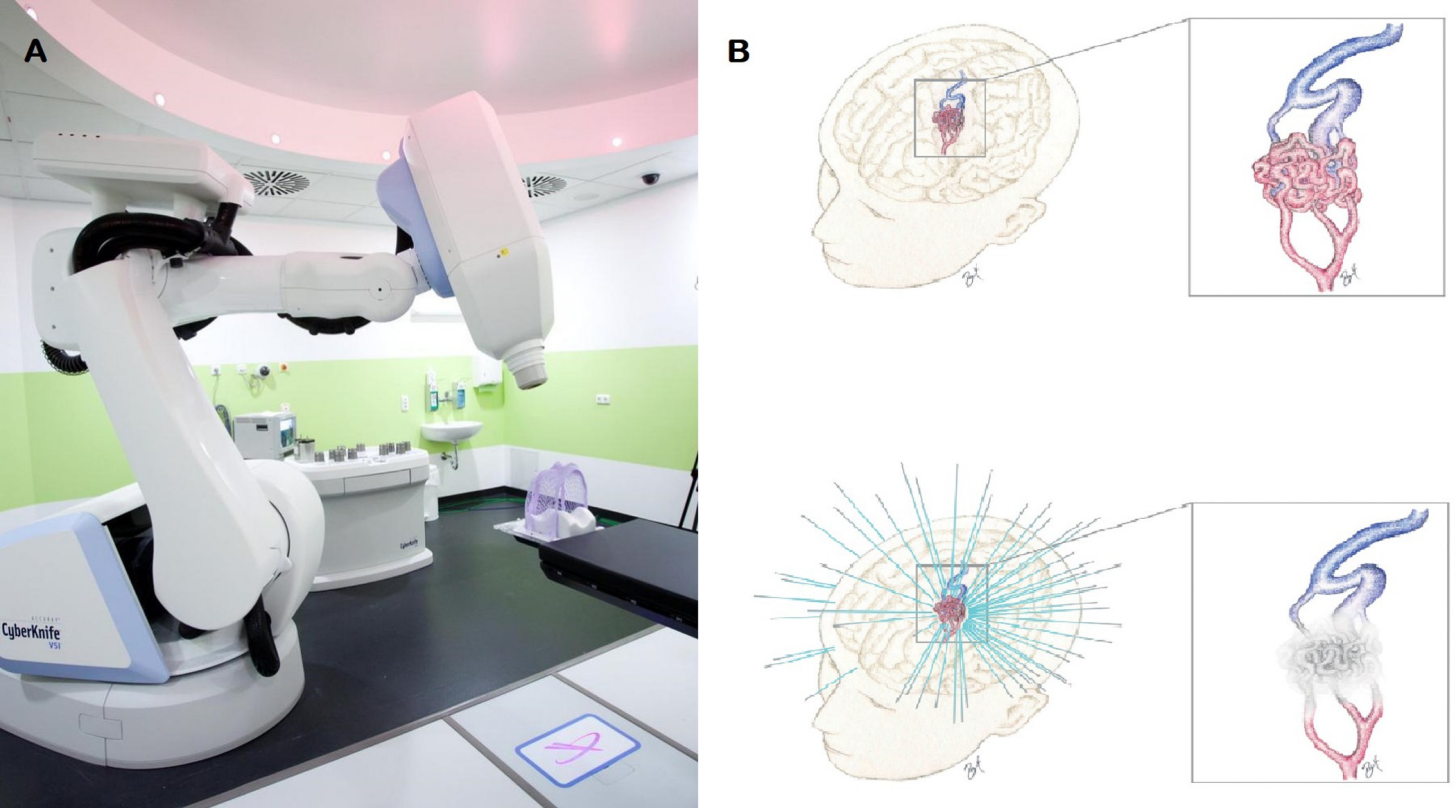
Illustration of stereotactic CyberKnife radiosurgery of an arteriovenous malformation. (a) CyberKnife machine used at our institution (b) Schematic illustration of the nidus of an intracranial arteriovenous malformation (AVM) before and after radiosurgical obliteration (credit: RX).

The CyberKnife System (Accuray, Inc., Sunnyvale, California) is an image-guided, radiosurgery system consisting of a 6 MV linear accelerator (LINAC) mounted on a robotic arm [9–12] (Fig 1). The advantage of CKRS in comparison to conventional LINAC based radiosurgery lies in two advanced technologies. Firstly, the robotic manipulator is highly maneuverable and can position and point the lightweight LINAC with immense precision (mean total radial error of 1.6mm, mean positioning error of 0.9 mm per axis). Secondly, real-time image guidance via radiographs acquired in regular intervals by two fixed X-ray fluoroscopes sis used to detect changes in positioning, and correct beam pointing in near real-team before delivery of radiation. [13] Compared to its predecessor, Gamma Knife radiosurgery, CKRS provides frameless non-isocentric irradiation and can thus create different dose distributions inside and outside the lesion [8–12,14].

Definitive data on CyberKnife radiosurgery (CKRS) alone or in combination with endovascular treatment, are limited [15–17]. This retrospective study aimed to analyze the data of CKRS-treated patients from two international centers to improve the current understanding of the safety and efficacy of CKRS for AVMs.

## Materials and methods

### Study design

We retrospectively analyzed clinical and radiographic data of 123 patients with AVMs of all SM grades treated between 2007 and 2018 at two academic institutions (Charité Universitaetsmedizin Berlin, Germany, and Università degli studi di Messina, Italy). The study was approved by the local ethics committees (EA1/233/18 and Comitato etico Messina Prot. 34/19).

### Image acquisition

#### Computed tomography planning/angiography

A computed tomography (CT) scan was obtained for radiotherapy planning (120 kV; ≥ 400 mAs; slice thickness: 0.75–1.0 mm; matrix: 512 × 512). To visualize cerebral arteries, 100 mL of iodinated contrast medium was injected intravenously (flow: 4 mL/s). Images were acquired in the arterial phase after a 24 s delay. The resulting three-dimensional CT angiography (CTA) was used for target volume contouring and dose calculation.

#### Magnetic resonance angiography

Magnetic resonance imaging (MRI) was performed prior to radiotherapy planning using a conventional 1.5 T or 3 T MRI scanner to generate high-resolution T1-weighted images and MR angiography (MRA). MRA was performed with a time-of-flight (TOF) sequence.

#### Digital subtraction angiography

Two-dimensional angiographic examinations were performed in advance using standard biplanar angiographic units. Selective four- or six-vessel digital subtraction angiography (DSA) was performed initially; additional views and rotational angiography for 3D DSA were obtained if required to identify the arterial feeders and for treatment planning.

### CyberKnife treatment planning

The selection of CKRS treatment was based on the consensus of a multidisciplinary board of neurovascular experts, including an interventional radiologist, a neuroradiologist, a neurosurgeon, and a radiation oncologist. CKRS treatment was planned using MultiPlan (Accuray Inc., Sunnyvale, CA) at both the institutions. The target volume was defined as the AVM nidus volume based on fused CTA images and the MRA TOF sequence and /or 3DRA.

Single-fraction doses of 14.0–22.0 Gy, depending on the nidus size and location, were administered to isodose lines of 70–85%.

### Follow-up

MRA was performed every six months for the first year, then every 12 months until the nidus was no longer recognizable. When obliteration was suspected on MRA imaging, DSA was performed for confirmation. The obliteration was classified as “complete obliteration” (CR), “partial obliteration” (PR), or “no change” (NC). CR was defined as complete obliteration of the nidus of the AVM on MRA. PR was defined as partial obliteration of the AVM with ≥25% decrease in the nidus size on contrast-enhanced MR scans. Obliteration was classified as NC when no signs of obliteration or decreased nidus size were evident. Treatment-associated effects were recorded, including radiographically confirmed perifocal edema or radiation necrosis requiring prolonged corticosteroid treatment.

### Data analysis

Clinical data were retrieved, including age at diagnosis, sex, modified Rankin Score (mRS), AVM location and hemisphere, SM grading [5], symptoms at initial diagnosis, date and symptoms at last follow-up, rebleeding occurrence, and the incidence of treatment-induced toxicity, including epileptic seizures or new-onset focal neurological deficits (hemiparesis, hypoesthesia, and cranial nerve deficits). In patients who underwent embolization, SM grades were recorded before and after embolization. Detailed data can be made available by the senior author upon request.

### Statistical analysis

Statistical analysis was performed using Statistical Package for the Social Sciences (SPSS) Statistics (version 25.0. Armonk, NY: IBM Corp.). Group comparisons were performed via the Mann-Whitney U test for metric data or chi-square test for categorical data. Logistic regression analysis was conducted to assess the influence of confounding variables on obliteration and complication rates. Kaplan-Meier-curves were generated to calculate the obliteration rates over time. The log-rank test was used for subgroup comparisons of obliteration times. Results were declared as significant for p-values < .05.

## Results

### Clinical and radiographic data

Between 2007 and 2018, 123 patients with AVMs were treated by CKRS. The median age at CKRS initiation was 39.8 years (range, 9.0–76.0) years. The ratio of males (62) to females (61) was balanced. The median mRS was 2 (range, 1–4). Initially, 68 (55.3%) patients presented with an intracerebral hemorrhage; the others were diagnosed with AVMs following complaints of headaches, epileptic seizures, and motor or sensory deficits. In some cases, diagnosis was incidental.

The median follow-up was 48.1 months (range, 3.6–132.0 months). In a subgroup of 59 patients whose follow-up exceeded four years, the median time was 61.2 months (range, 48.0–132.0 months).

Table 1 summarizes the clinical characteristics of the entire cohort and the two subcohorts.

**Table 1:** Clinical characteristics of the patient cohort and subcohorts.

		Entire cohort	Berlin cohort	Messina cohort
Number of patients		123	39	84
Median age (range) in years		39.8 (9–76)	37.6 (9–74)	44.5 (14–76)
Male: female ratio		62: 61	23: 16	39: 45
Symptoms	hemorrhage	68 (55.3%)	17 (43.6%)	51 (60.7%)
	other	55 (46.7%)	22 (56.4%)	33 (39.3%)
Side	right	52 (42.3%)	17 (43.6%)	35 (41.7%)
	left	58 (47.2%	19 (48.7%)	39 (46.4%)
	median	13 (10.6%)	3 (7.6%)	10 (11.9%)
Location	frontal	31 (25.2%)	8 (20.5%)	23 (27.4%)
	temporal	21 (17.1%)	6 (15.3%)	15 (17.9%)
	parietal	22 (17.8%)	8 (20.5%)	14 (16.7%)
	occipital	7 (5.6%)	1 (2.6%)	6 (7.1%)
	other	42 (34.1%)	16 (41.0%)	26 (30.9%)
Spetzler-Martin grade	I	11 (8.9%)	1 (2.6%)	10 (11.9%)
	II	4 (35.7%)	3 (7.7%)	41 (48.8%)
	III	37 (30.1%)	16 (41.0%)	21 (25.0%)
	IV	24 (19.5%)	12(30.8%)	12 (14.3%)
	V	7 (5.7%)	7 (17.9%)	0
Embolization		54 (43.9%)	12 (30.7%)	42 (50.0%)

Based on SM classifications, 11 patients had grade 1 lesions, 44 had grade 2 lesions, 37 had grade 3 lesions, and 24 and 7 had grade 4 and 5 lesions, respectively. Embolization was performed before radiosurgery in 54 patients.

### Technical treatment parameters

All patients were treated with a single radiation fraction, with a median prescription dose of 20.0 Gy (range, 14.0–22.0 Gy). The median prescription isodose line was 80% (range, 70%–85%), and the median treatment duration was 61 minutes (range, 38–116 minutes). The treated volume was 0.2–27.2 cm^3^, with a median of 3.4 cm^3^.

### Obliteration rates

**Fig 2:**
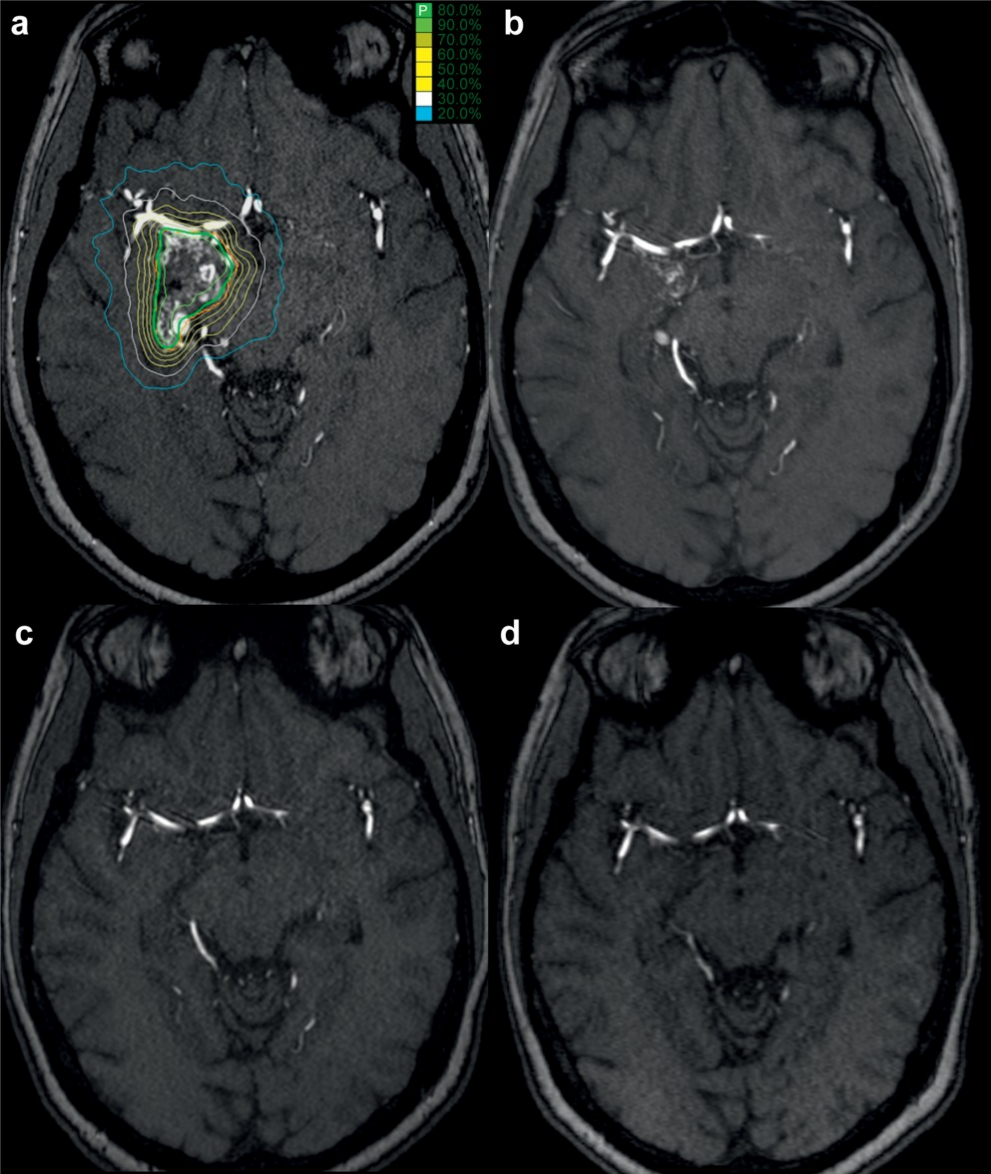
Exemplary obliteration process after CyberKnife radiosurgery. An example of the obliteration process for one patient with Spetzler-Martin grade IV arteriovenous malformation (AVM). The images show the CT scan with the CyberKnife treatment plan (red: target, green: prescribed isodose) a MR angiography (a), the MR angiography scans 8 months after treatment showing partial obliteration (b), and MR angiography scans of the same area two years (c) and five years (d) after radiosurgery, demonstrating complete obliteration of the AVM.

Five of 123 patients were lost to follow-up and excluded from the obliteration analysis. In the remaining 118 cases, complete obliteration was observed in 67 cases; in 44 (65.7%) of these, DSA confirmed complete obliteration. A partial response was seen in 31 cases, and no change was observed in 20 (Fig 2). In the cases with DSA-confirmed complete obliteration, the median time was 24.0 months (range, 12.0–72.0 months). An example of the obliteration process in a patient treated with CKRS over a five-year follow-up period is shown below (Fig 2).

**Fig 3:**
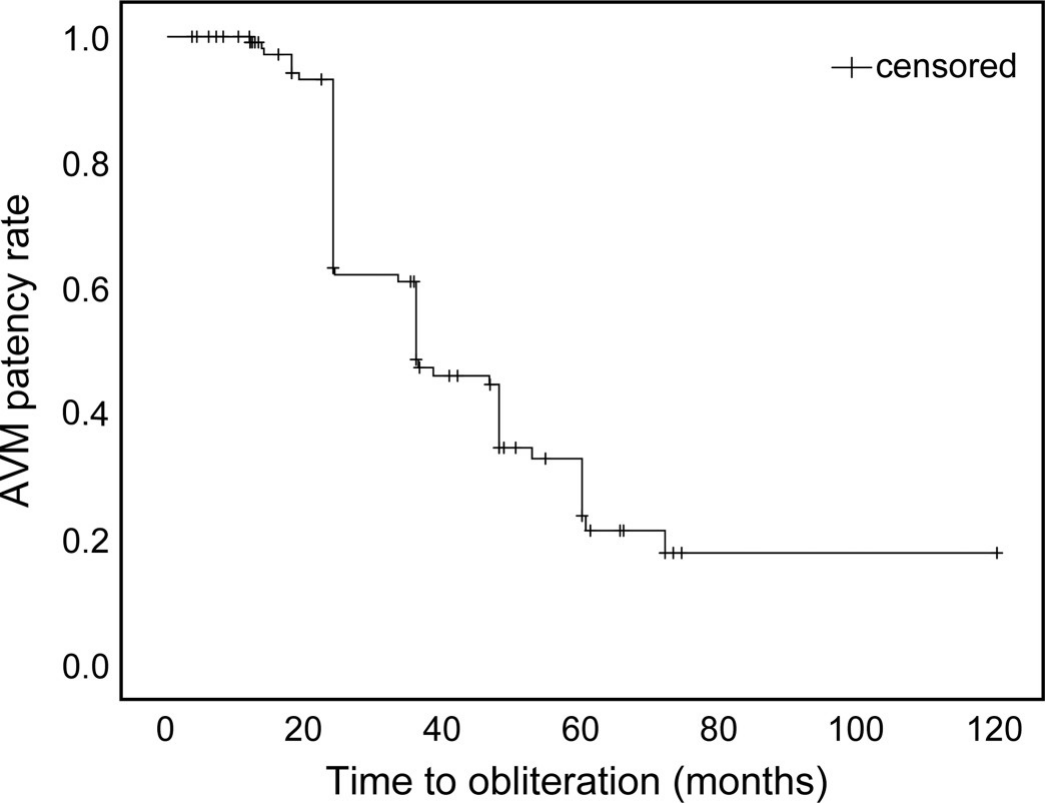
Time to obliteration after CyberKnife radiosurgery–Kaplan-Meier curve. Kaplan-Meier curve of the time (in months) to the obliteration of arteriovenous malformations (AVMs) in patients treated via CyberKnife radiosurgery. Obliteration is observed between 24 and 48 months after treatment in the majority of patients.

In the subgroup of 59 patients whose follow-up exceeded four years, complete obliteration was achieved in 43 (72.8%); a partial response and no change were each observed in seven patients (11.9%). Kaplan-Meier analysis illustrated that most experienced obliteration after two to four years of treatment (Fig 3).

### Embolization and AVM downgrading

**Fig 4:**
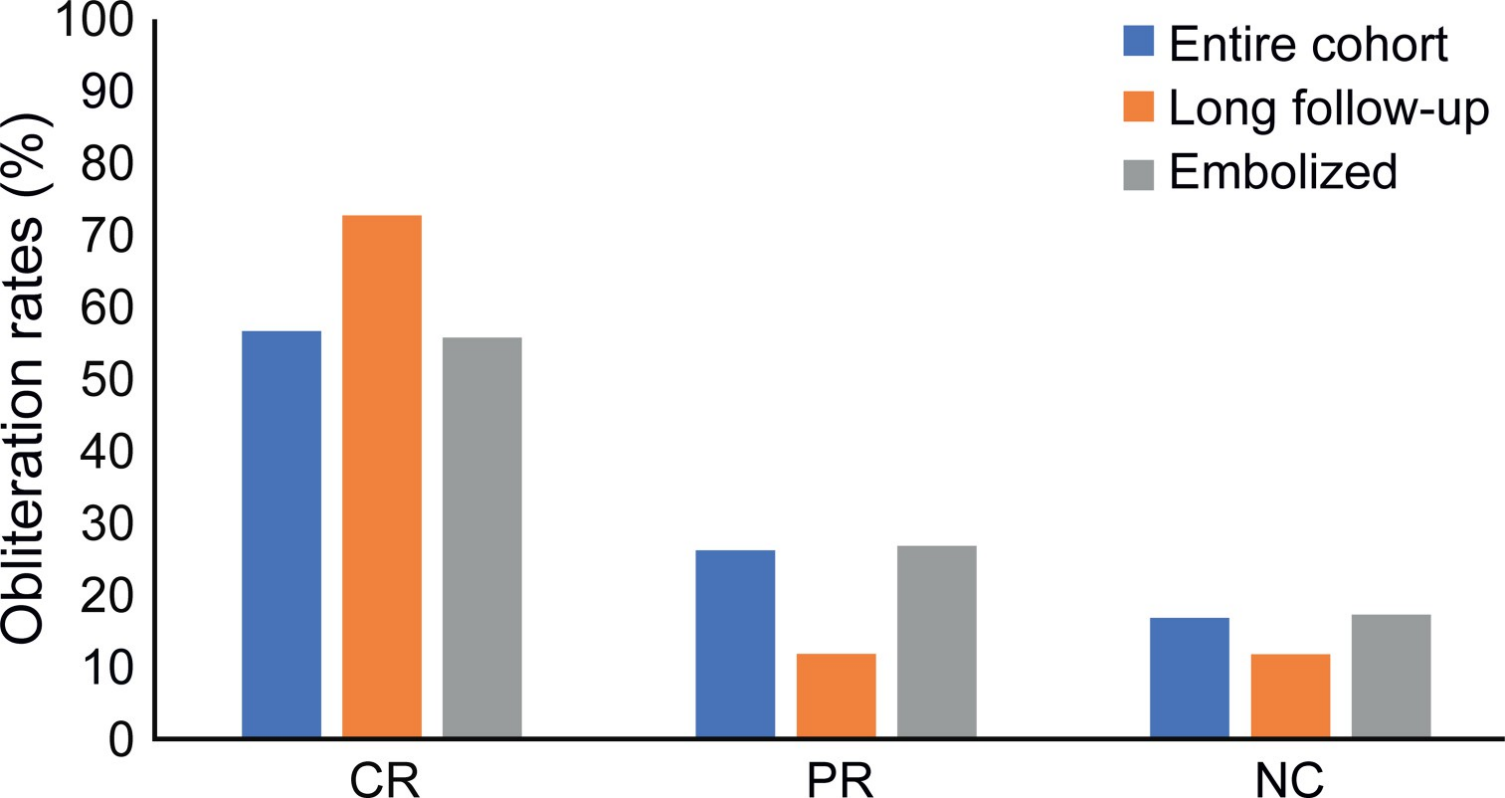
Obliteration rates. Obliteration rates in the entire cohort (blue), in the subcohort of patients with a follow-up duration exceeding four years (orange) and in previously embolized patients (grey). CR = complete response; PR = partial response; NC = no change.

Embolization was performed in 54/123 cases (43.9%) prior to initiating CKRS, resulting in downgrading in 23 cases (42.6%); in two cases, the SM grades were reduced by two (3.7%). Two patients were lost to follow-up. In the remaining subgroup of 52 embolized patients, complete and partial obliteration were achieved in 29 and 14 cases, respectively. No change was observed in nine cases (Fig 4).

In the subgroup of 66 patients without embolization, 38, 17, and 11 exhibited complete or partial obliteration, and no change, respectively. The complete obliteration rate did not significantly differ between the embolized and non-embolized subgroups.

### Differences in clinical outcomes in “high-grade” versus “low-grade” AVMs

**Fig 5:**
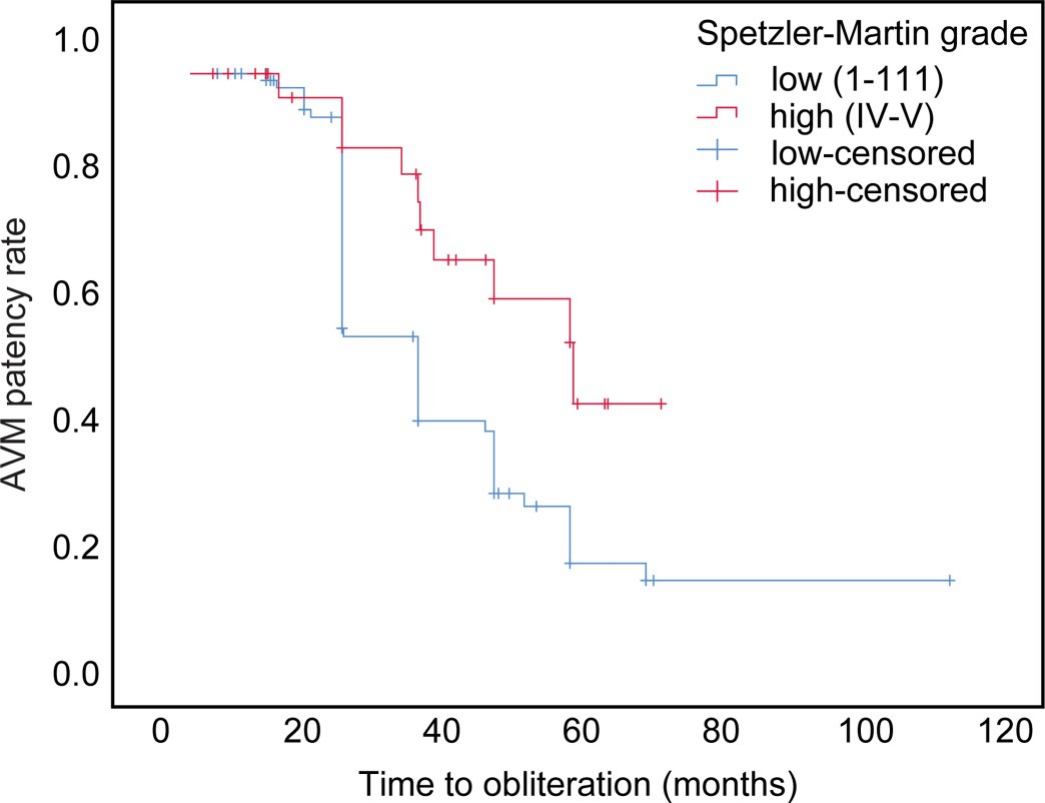
Time to obliteration after CyberKnife radiosurgery in patients with “low-grade” vs. “high-grade” arteriovenous malformation. Kaplan-Meier curve of the time to obliteration (in months) in the subgroups of patients with “low-grade” (Spetzler-Martin grade I–III) vs. “high-grade” (Spetzler-Martin grade IV–V) arteriovenous malformations (AVMs) treated via CyberKnife radiosurgery. The curve shows a significant difference in the times to obliteration between the two groups (p = .036).

We classified the subgroup with SM grade I-III lesions as “LG” (“low-grade”) (73.9%) and the subgroup with SM grade IV-V lesions as “HG” (“high-grade”) (26.0%) (Table 2). We observed a statistically significant difference in local control of the lesion (complete versus partial or no change; p = .005, chi-square test), with a mean time to obliteration of 32.5 and 41.5 months in the LG and HG subgroups, respectively (p = .036, log-rank test; Fig 5).

**Table 2:** Differences in clinical outcomes in “high-grade” vs. “low-grade” AVMs treated with CKRS.

	“high-grade” AVMs	“low-grade” AVMs
Spetzler-Martin grades	IV–V	I–III
Number of patients	31	92
Complete response (CR)	10 (34.5%)	57 (64.0%)
Partial obliteration (PR)	11 (37.9%)	19 (21.3%)
No change (NC)	8 (27.6%)	12 (13.4%)
Patients lost for follow-up	2 (6.5%)	3 (3.2%)

In the LG subgroup, a significantly higher prescribed dose had been administered, with means of 19.4 Gy and 17.7 Gy in the LG and HG subgroups, respectively (p = .001, Mann-Whitney U test).

### Rebleeding, complications, and treatment-induced toxicity

Five patients (4.2%) experienced a bleeding incident during follow-up; four had bleeds from previously irradiated AVMs, and one experienced intracerebral hemorrhage distant from the AVM. Four patients (3.4%) died during follow-up, with the cause of death related to the treated AVMs in two cases (1.7%).

Therapy-associated complications were experienced by 26 patients. Six patients experienced epileptic seizures after radiotherapy and were treated sufficiently with antiepileptic medication. Edema was observed in 15 patients, with severe edema requiring prolonged corticosteroid (dexamethasone) treatment in two patients. Two cases of radiation necrosis were observed and one incident of sinus vein thrombosis. Overall, 10 patients suffered from hemiparesis, either due to previous bleeding or intervention, or a new onset resulting from edema and radiation effects. Four of 123 treated patients exhibited new-onset deficits following radiosurgery, whereas 92 remained symptom-free throughout follow-up.

In the LG subgroup, 14/89 patient experienced complications, and 12/29 patients in the HG subgroup experienced edema, seizures, or other adverse effects. The incidence was significantly higher in the HG than in the LG subgroup (p = .004, chi-square test).

### Factors predicting outcomes and complication rates

Logistic regression analysis revealed that age (p = .018, and SM grade (p = .041) were independent predictors of obliteration, with higher grades and older age associated with a lower probability of obliteration. No statistically significant association was observed between obliteration and initial bleeding (p = .481) or prescription dose (p = .063) (Table 3). The logistic regression analysis revealed that only the treated volume was independently associated with the occurrence of complications (p = .009) (Table 4).

**Table 3:** Influence of age, hemorrhagic onset, prescription dose and Spetzler-Martin score on obliteration.

	Regression coefficient	Standard deviation	Wald	p-value	Odds Ratio	95% confidence interval	
Age	-.031	.013	5.617	.018*	.969	.945	.995
Hemorrhagic Onset (yes)	-.290	.412	.497	**.519**	.748	.334	1.677
Prescription dose	.285	.153	3.449	**.076**	1.329	.841	1.795
Spetzler-Martin grade	-.482	.236	4.191	.041*	.617	.389	.980

Test: Logistic regression,

* p < .05 is considered statistically significant.

**Table 4:** Influence of age, hemorrhagic onset, prescription dose and Spetzler-Martin score on the rate of complications.

	Regression coefficient	Standard deviation	Wald	p-value	Odds Ratio	95% confidence interval	
Age	-.005	.016	.104	.748	.995	.964	1.026
Hemorrhagic Onset (yes)	-.484	.508	.907	.341	.616	.227	1.669
Prescription dose	.087	.178	.240	.624	1.091	.769	1.548
Spetzler-Martin grade	.277	.279	.984	.321	1.319	.763	2.279
Volume	.126	.048	6.915	.009*	1.135	1.033	1.246

Test: Logistic regression,

* p < .05 is considered statistically significant.

## Discussion

Although microsurgical resection remains the standard for treating intracranial AVMs, deep-seated or higher-grade lesions remain major neurosurgical challenges.

The 123 patients treated by CKRS for AVMs demonstrated satisfactory outcomes, with complete and partial obliteration achieved in 56.8% and 26.3% of cases, respectively; in those with follow-up times exceeding four years, the rates were 72.8% and 11.9%, respectively. The rate of complete obliteration was significantly higher in the LG than in the HG subgroup, and only 4.2% of the patients experienced recurrent hemorrhage or treatment-related toxicity.

Our cohort mirrors the demographic characteristics of previously published series in terms of the median age, male-to-female ratio, and distribution of SM grades [15]. Similarly, approximately 50% of patients in our cohort initially presented with intracerebral hemorrhage [8]. The radiation doses and other radiometric parameters were comparable to those reported in previous studies, including the Gamma Knife series [16,18–20]. Our study was the second largest series of patients with AVMs treated with CKRS; to date, only one study has reported the outcomes in a larger cohort [8]. In the subgroup with follow-up time exceeding four years, the combined rate of MRA-and DSA-confirmed CR was 72.8%; this finding was consistent with the obliteration rates reported in other studies [7,8,15,16,20] and reflected the obliteration rate of 71.5% previously reported in 279 patients with AVM treated with CKRS with 36 months of follow-up [8]. Additionally, our results compared well with those reported in a cohort of 173 patients with low-grade AVMs wherein an obliteration rate of 76% was achieved, while a higher obliteration rate (83%) was achieved after repeated SRS; this suggested that re-irradiation could be considered as an option for partially occluded lesions after at least four years of follow-up [21].

Age and SM grade were independent predictors of treatment responses in our cohort. SRS is more effective in younger patients, possibly because vascular aging causes microvascular remodeling, which negatively influences radiation-induced obliteration [22]. This inverse association between age and obliteration forms the clinical basis for including age in radiosurgical AVM scoring [23]. The SM grade, particularly AVM volume, is the most important factor influencing SRS outcomes [20,21,24].

About 44.0% of the patients received endovascular treatment before radiosurgery, leading to AVM downgrading in 42.6% of patients and facilitating radiosurgical treatment by reducing target volumes and lesion complexity (i.e., by reducing AVM compartmentalization or obliterating portions lying within critical brain locations to minimize direct irradiation). Our results are in contrast with those of previous series [7,8] showing that embolization adversely effects final obliteration rates, possibly due to suboptimal dose calculation due to the high-Z value of embolizing materials and segmentation of the nidus in multiple and distant compartments. In our series, this was avoided by performing embolization only as a propaedeutic procedure to radiosurgery, aimed at AVM downgrading while avoiding nidus fragmentation.

Recurrent intracranial hemorrhage after AVM irradiation presents the most threatening complication. In a large multi-center cohort of 2,320 AVMs, a 1.2% incidence of rebleeding was reported [15], and similar rates have been reported for Gamma Knife radiosurgery [25,26], consistent with our own data. In our population, of the five patients (4.1%) experiencing a hemorrhage after SRS, two (1.6%) died from the consequences of these bleeding events. The incidence of recurrent hemorrhage did not differ substantially from the natural course of the disease. Common adverse effects after high-dose SRS include radiation necrosis, which usually occurs within the first 12–18 months post-irradiation, and post-treatment symptomatic edema, potentially causing epileptic seizures, headaches, intracranial hypertension, or focal neurological symptoms. In our cohort, only about 5.1% of patients experienced post-radiotherapy seizures, and 12.7% exhibited symptomatic treatment-induced edema; only three patients (2.5%) required prolonged steroid therapy, similar to the adverse effect incidences reported for other cohorts using similar doses [8,21,27].

The treatment of lesions of SM grades IV and V is a matter of intense debate [28–31]. In this subgroup, the obliteration rate after endovascular downgrading followed by SRS was significantly lower (34.5% vs. 64.0%; p = .005), with a rate of persistent neurological deficits of 24.1% (7/29 patients) and a higher rebleeding rate (4/29, 13.8%). These results may contribute to the debate pertaining to the best option for treating higher-grade AVMs. Microsurgery, if feasible, should still be considered for lesions with an acceptable risk-benefit profile.

In contrast to LINAC based radiosurgery as well as Gamma Knife radiosurgery, the CKRS method proved to be less traumatic, as no head fixation is required, and can be performed as an outpatient procedure. Planning image acquisition and treatment planning can be performed in different places and times, thus reducing the actual treatment time for the patient [7]. While obliteration rates between LINAC based radiosurgery vs. Gamma Knife radiosurgery have not shown significant difference [32], the high precision of CKRS ultimately may lead to better obliteration. Due to its nonisocentric irradiation geometry and homogenous dose distribution, the CKRS technique additionally decreases the risk of hemorrhage [7].

While CKRS proved to have many advantages over conventional methods, we encountered a few institutional challenges regarding the application of this therapy. First of all, financiation is secured only for a subset of the patients and applications for cost coverage can be tedious. In our instituition, CKRS can only be performed in an outpatient setting. In our experience, single-dose irradiation more likely leads to initial headache and nausea than conventional methods. We addressed this issue by prophylactically administering dexamethasone for a few days. Therefore, we generally recommend CKRS for younger patients in a good general condition, who are more likely to tolerate this outpatient treatment.

This study had some limitations. The retrospective nature limits the conclusions, and the multi-centric design may have resulted in inconsistencies in patient selection. In cohort 1, only large or complex AVMs were treated radiosurgically, whereas in cohort 2, the CKRS technique was also applied to AVMs of lower SM grades. However, as the treatment characteristics are comparable among both institutions, our analysis of this large international AVM cohort treated with CKRS remains of interest. All patients were followed up with MRA imaging. When AVM obliteration was evident by MRA, patients underwent DSA for confirmation; a proportion of patients (16.1%) are still waiting for a definitive confirmation of obliteration. Lastly, the 48-month median follow-up time might have been inadequate for assessing some outcome measures after CKRS, including later obliterations. To address this limitation, we emphasized the analysis of the patient subgroup with follow-up times exceeding four years.

In conclusion, CKRS achieved obliteration rates in line with the literature, with low toxicity and a relatively rare incidence of rebleeding. While microsurgical resection remains the first choice for lesions with an acceptable risk-benefit profile, CKRS appears to be a valid therapeutic approach. To confirm our findings, a longer follow-up time and prospective data would be desirable.
